# The temporal dynamics of visual working memory guidance of selective attention

**DOI:** 10.3389/fnbeh.2014.00345

**Published:** 2014-09-26

**Authors:** Jinfeng Tan, Yuanfang Zhao, Shanshan Wu, Lijun Wang, Glenn Hitchman, Xia Tian, Ming Li, Li Hu, Antao Chen

**Affiliations:** ^1^Key Laboratory of Cognition and Personality of Ministry of Education, Faculty of Psychology, Southwest UniversityChongqing, China; ^2^Department of Psychology, University of Nebraska-LincolnLincoln, NE, USA

**Keywords:** working memory, biased competition model, event-related potential (ERP), sLORETA, alpha-band rhythm

## Abstract

The biased competition model proposes that there is top-down directing of attention to a stimulus matching the contents of working memory (WM), even when the maintenance of a WM representation is detrimental to target relevant performance. Despite many studies elucidating that spatial WM guidance can be present early in the visual processing system, whether visual WM guidance also influences perceptual selection remains poorly understood. Here, we investigated the electrophysiological correlates of early guidance of attention by WM in humans. Participants were required to perform a visual search task while concurrently maintaining object representations in their visual WM. Behavioral results showed that response times (RTs) were longer when the distractor in the visual search task was held in WM. The earliest WM guidance effect was observed in the P1 component (90–130 ms), with match trials eliciting larger P1 amplitude than mismatch trials. A similar result was also found in the N1 component (160–200 ms). These P1 and N1 effects could not be attributed to bottom-up perceptual priming from the presentation of a memory cue, because there was no significant difference in early event-related potential (ERP) component when the cue was merely perceptually identified but not actively held in WM. Standardized Low Resolution Electrical Tomography Analysis (sLORETA) showed that the early WM guidance occurred in the occipital lobe and the N1-related activation occurred in the parietal gyrus. Time-frequency data suggested that alpha-band event-related spectral perturbation (ERSP) magnitudes increased under the match condition compared with the mismatch condition only when the cue was held in WM. In conclusion, the present study suggests that the reappearance of a stimulus held in WM enhanced activity in the occipital area. Subsequently, this initial capture of attention by WM could be inhibited by competing visual inputs through attention re-orientation, reflecting by the alpha-band rhythm.

## Introduction

The human cognitive system cannot process every input because of its limited capacity. Selective attention plays a critical role in human information processing, preventing information overload by allocating limited resources to the most critical and relevant aspect of information and inhibiting those irrelevant to current goals. The influential biased competition model of visual selection proposes that the neural representations of different objects in the scene are mutually inhibitory, competing for access to higher level processing, with object selection being controlled by the preactivation of the neural channels responsive to a particular relevant object (Desimone and Duncan, 1995). Within this framework, top-down control signals from object representations in working memory (WM) act to bias the competition for attention to favor items that match the “template” held in memory (Duncan and Humphreys, 1989).

Evidence for the top-down bias from WM has been shown in a body of behavioral data on visual search based on the delayed-match-to-sample task paradigm (Downing, 2000; Soto et al., 2005; Soto and Humphreys, 2008, 2009; Pan et al., 2009). These studies typically compare a condition where a memory item reappears as an irrelevant distractor in a search task to a condition where a distractor of a search display is unrelated to the memorized item. The critical finding is that a distractor matching the content of memory could more severely disturb a target search than an unrelated distractor, as indexed by increased search RTs. Of note, even when WM guidance is detrimental to performance, the guidance still happens, suggesting that WM automatically or involuntarily guides attention. Moreover, a number of studies have recently demonstrated that reentrant feedback from WM can affect early stage of perceptual processing (Soto et al., 2010; Pan et al., 2012). For example, Soto et al. advocated a perceptual enhancement effect of visual WM on attentional processing. In this account, objects in visual memory increase the perceptibility of items that share features with the remembered objects. Pan et al. (2012) also demonstrate that unconscious processing of a stimulus property can be enhanced when there is a match between the contents of WM and the stimulus presented in the visual field. These findings argue against a dominant role of spatial attention over selection based on object properties (Theeuwes and Van der Burg, 2007), and suggest that the perceptual consequences of maintaining object information in WM may operate sufficiently and rapidly to influence perceptual discrimination.

In other studies, though, effects of WM on visual selection have not always been observed. For example, Downing and Dodds (2004) showed participants two shapes, one of which was the target in a subsequent search display, and the other a memory stimulus that had to be remembered for a later report. They showed that the memory stimuli did not capture attention when they reappeared in a search display. Similar findings were also reported in Woodman and Luck’s (2007) study. These results suggest that, at least under certain conditions, information in WM that is irrelevant for current behavior can be shielded from the relevant task at hand, or it might even be used to guide search away from irrelevant distractors.

More converging evidence about the underlying mechanism of whether and how WM influences attention can be found in neurophysiological studies. Single-cell recordings in monkeys have demonstrated that a match between a stimulus and a WM representation is associated with increased responses in the inferior and medial temporal cortex (Chelazzi et al., 1993, 1998). Other neuroimaging studies using similar paradigms with human participants suggest that the reappearance of a stimulus held in WM enhanced activity in the superior frontal gyrus, midtemporal, and occipital areas that are known to encode the prior occurrence of stimuli (Soto et al., 2007, 2011; Grecucci et al., 2010).

Moreover, the event-related potentials (ERPs) technique with high temporal resolution is a useful method to investigate when the WM content exerts its effect on attention. Mangun et al. (1993) found that the amplitudes of the P1 and N1 components were considerably enlarged for targets presented to the attended spatial location relative to targets in unattended locations (see also Luck et al., 1993; Johannes et al., 1995; Wijers et al., 1997; Luck and Hillyard, 2000). As the early P1 and N1 component are sensitive to spatial attention, they may index spatial memory-based attentional guidance. However, the story seems different when the guidance effect is driven by object representations in WM. Kumar et al. (2009) reported that these early components were not influenced by WM contents when participants were asked to hold colored geometric patterns in memory. By contrast, the amplitude and latency of the N2pc were modulated by WM guidance, indicating an early capture of attention by the stimuli matching the content of WM. Telling et al. (2010) asked participants to search for a target in a four-object display that could include a semantically WM related distractor. Neither the P1 nor the N1 component showed any difference in activity across the target status and distractor condition. These results indicate that the differences between targets and distractors are not sufficient to influence early perceptual processing.

Although several lines of evidence suggest a role for WM in top-down attentional guidance, these ERP studies in which researchers tested the time courses of how WM representations bias the deployment of attention have yielded diverse findings. We suspect that one problem may be due to a lack of strict controls on experimental materials. For example, in a typical delayed-match-to-sample task, participants are usually asked to memorize a simple visual stimulus (e.g., color, shape, or both) for a subsequent memory test. However, the simple visual stimuli may weaken the WM guidance effect. Specifically, it would be easier for subjects to maintain these stimuli with semantic rehearsal in WM (such as red triangle). It is known that the guidance effect of verbal memory is generally weaker relative to visual memory (Olivers et al., 2006) and early semantic distractor interference takes around 200 ms (Telling et al., 2010). Therefore, the N2pc modulation observed by Kumar et al. (2009) may not index the earliest ERP modulation of the visual WM guidance effect. On the other hand, as the aforementioned studies always use ordinary life objects (e.g., fish) and geometric shapes as experiment materials, it is possible that participants’ familiarity with materials contributes to the verbal encoding of contents in WM, which undermines the effect of object memory-based attentional guidance.

In response to the potential drawbacks of the previous studies, in the present experimental design, we made two improvements to provide further evidence of the top-down guidance of attention. On the one hand, we controlled the type of experimental material, in order to obtain a pure visual WM. There are two methods which can be employed to prevent participants from verbally recoding visual information. Firstly, participants were asked to carry out an articulatory suppression task prior to the presentation of the memory item (Downing, 2000; Downing and Dodds, 2004). Secondly, novel shapes, which are difficult to verbalize, were used as experimental materials. This type of visual stimuli has been successfully used in prior memory studies (Jiang et al., 2000; Olson et al., 2008). As the WM effect on subsequent search has been shown to be reduced when a verbal suppression task was included (Soto and Humphreys, 2008), the latter method was applied in the current research and irregular forms were designed as memory materials. Moreover, to optimize observers’ performances in the subsequent memory test, the meaningless memory pictures would force them to adopt deeper visual memory representations. By doing so, a strong guidance effect might be observed during the later visual search task. On the other hand, different from the previous search displays where tilted and vertical lines embedded in geometrics were utilized (see Soto et al., 2008 for a review), our stimulus display consisted of a centrally presented imaginary circle of six letters with a peripheral meaningless picture as the distractor presented to the left or right of the circle. The subjects were instructed to search the letter circle for a target letter and ignore the peripheral meaningless picture (distractor). Consequently, the clear physical distinction between target search and distractor could reduce the interference from neural letters in circle to distractor, which makes WM guidance exert it best effect and perhaps influence the early perceptual processing stage.

In the present study, we utilized ERPs to investigate the early ERP correlates of the visual WM guidance effect. In addition to traditional ERP waveform analysis, we also applied the source localization analysis with sLORETA method (Pascual-Marqui, 2002) to find the location of such guidance effect. In addition, time-domain electroencephalogram (EEG) signals were further transformed to time–frequency domain event-related spectral perturbation (ERSP) activities. Time–frequency analysis is believed to reflect some kind of stimulus-induced ongoing EEG activity, which carries important information about cognitive processing that is usually averaged out as background noise in the traditional ERP analysis (Makeig et al., 2004). Previous studies have suggested that event-related synchronization (ERS) in the alpha-band (8–13 Hz) is often related to top-down inhibitory control processing (Klimesch et al., 2007; Jensen and Mazaheri, 2010). According to the biased competition model, we expected to find slower responses when the distractor of the visual search task matched (rather than mismatched) the memory item. If visual distractors influence early perceptual processing, changes in P1 and N1 amplitudes would be expected. Moreover, we speculated that oscillations in the alpha-band frequency range may be the potential neural oscillatory correlates of WM guidance.

## Materials and methods

### Participants

Forty right-handed healthy undergraduates, with normal or corrected-to-normal vision, were recruited from Southwest University as paid participants. 18 participants (aged 19–26, 9 males) were assigned to the WM group and 22 participants to the mere repetition group. In the mere repetition group, data from two participants were excluded because of technical problems, and data from three participants were also excluded from further analyses because of excessive eye blinks. The remaining 17 participants (9 male) were between 19 and 26 years of age. Participants were free from neurological diseases and reported no history of psychoactive medication use. Informed consent was obtained from all the participants, who were unaware of the purpose of the present experiment. The study was approved by the local ethics committee.

### Stimuli and procedure

**Figure 1:**
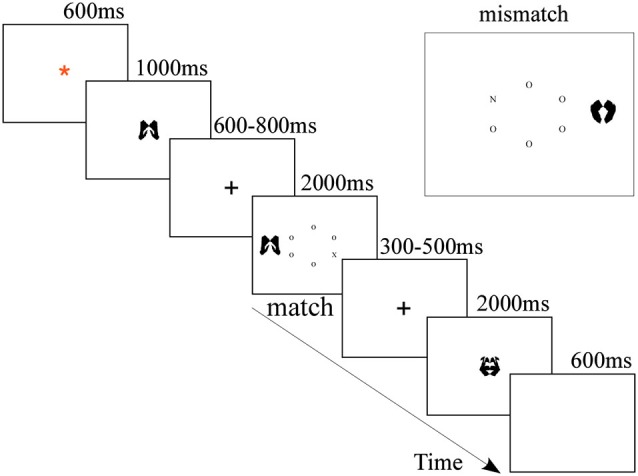
**Sequence and timing of stimulus events presented on each trial of the WM group**.

E-prime Software (Psychology Software Tools, Inc. Pittsburgh, PA) was used to present the stimuli and record the behavioral responses of the participants. The stimuli (letters and images) were displayed on a 17″ computer screen placed about 60 cm away from the participants. As depicted in Figure 1, the stimuli were black and presented on white background. Trials began with a fixation (a red asterisk) in the center of screen for 600 ms. Then, the cue (the memory item) was displayed at the fixation location for 1000 ms, during which participants had to memorize the image cue for the subsequent memory test. After a 600–800 ms blank screen, the visual search task began and at longest lasted for 2000 ms, during which the participants needed to conduct a visual search and respond accordingly. Finally, the second fixation (a cross) jittered for 300–500 ms, followed by a memory probe image for 2000 ms, during which participants completed the memory test. In the mere repetition group, the experiment design was the same as the WM group, except that the cue appeared twice, the first time for 150 ms and the second time for 700 ms, with a blank interval of 150 ms between them (Soto et al., 2007; Kumar et al., 2009). Participants were instructed to perceptually compare the two instances of the cue and to withhold their response to the search display whenever the second presentation of the cue differed from the first presentation (20% likelihood throughout the experiment).

In the visual search task, participants were asked to search the letter circle for a target letter (either X or N) as quickly as possible. The stimulus display consisted of a centrally presented 1.41° radius imaginary circle of six letters (each subtending 0.15° by 0.15°), plus a peripheral meaningless picture (each subtending 3.1° by 3.1°) as the distractor, which was presented 1.75° to the left or right of the center of the circle. The nontarget letters in the circle were all Os placed randomly around the circle. Participants would press the “1” key of the keyboard if the target was N using their left middle finger and press the “0” key of keyboard if the target was X using their right middle finger. The visual search trial terminated once a response was made. On half of the visual search trials, the distractor matched the cue; on the other half, it did not. In the WM group, participants were instructed to hold the cue image in memory while performing the visual search task. In the mere repetition group, participants should not make a response to the search display when the two cues were different. They were explicitly instructed to ignore the distractor and respond as quickly as they could while not sacrificing accuracy.

Upon the appearance of the probe trials, in the WM group, the participants were instructed to indicate whether or not the probe image was the to-be-remembered image presented at the start of the trial. Subjects indicated either a same or different response by pressing the “same” (“Q”) or “different” (“O”) keys on the computer keyboard with the index fingers of their left hand and right hand, respectively. On half of the trials, the memory test was identical to the memory set; on the other half, it was not. The memory test terminated once a response was made. In the mere repetition group, participants should not make any response.

In the WM group, each participant completed 192 trials in 4 blocks. After each block of 48 trials, the participants had a short break (1 min), during which they were told to relax. In the mere repetition group each participant completed 240 trials in 4 blocks. After each block of 60 trials, the participants had a short break (1 min). All task parameters including target position, target identity, distractor condition, and response-sides were pseudo-randomized and counterbalanced across subjects. One training block of 12 trials was run prior to the start of the main experiment.

### ERP recoding

Brain electrical activity was recorded from 64 scalp sites using tin electrodes mounted on an elastic cap (Brain Products GmbH, Germany). All channels were referenced online to a channel located on FCz. They were also re-referenced offline to represent recording with respect to the average of the left and right mastoids. The vertical electrooculogram (EOG) was recorded with electrodes placed below the right eye, while the horizontal EOG was recorded with electrodes placed on the right side of the right eye. Inter-electrode impedance was maintained below 5 kΩ. The EEG and EOG were filtered using a 0.01–30 Hz band pass filter, and continuously sampled at 500 Hz/channel for offline analysis. Eye movement artifacts (blinks and eye movements) were rejected offline. Trials with EOG artifacts (mean EOG voltage exceeding ±80 μV) and those contaminated with artifacts because of amplifier clipping, bursts of electromyography activity, or peak-to-peak deflection exceeding ±80 μV were excluded from averaging.

### Data analysis

#### Behavioral analysis

Behavioral analyses examined the effects of search RTs, search accuracy and memory accuracy using two-way ANOVAs, that examined group (WM, mere-repeat) × distractor item (match, mismatch), and group was a between-subject factor. In WM group, only trials in which the subjects were correct on the memory test task were included in the accuracy analysis of the visual search task, and only the data of trials where both search responses and memory test responses were correct received the statistical analyses of visual search task RTs. In the mere repetition group, only correct responses were involved in RT analyses.

#### Electrophysiological analysis

Electrophysiological analyses examined the P1 and N1 components in the stimulus-locked waveform. In the WM group, only trials with correct responses for both tasks were used in the analyses. The averaged epoch for the ERP elicited by the visual search trials was 600 ms, including 400 ms post-stimulus and 200 ms pre-stimulus. These ERP waveforms were collapsed into match and mismatch waveforms, averaged cross the relationship between the cue and the distractors in search trials. Contralateral waveforms were constructed by averaging the left hemisphere electrodes for right hemifield distractors and right hemisphere electrodes for left hemifield distractors. Ipsilateral waveforms were constructed by averaging the right hemisphere electrodes for right hemifield distractors and left hemisphere electrodes for left hemifield distractors (Zhang and Luck, 2009; Sawaki and Luck, 2011). Based on previous research (Kumar et al., 2009; Pratt et al., 2011; Wang et al., 2012) and the scalp topography distributions of the difference waves in the present study, the following scalp regions-of-interest (ROIs) and time windows were defined. We chose the left occipito-parietal (PO3, PO7, and O1) and right occipito-parietal (PO4, PO8, and O2) scalp regions. We defined the time windows of P1 (90–130 ms) and N1 (160–200 ms) in the WM group and P1 (80–120 ms) and N1 (150–190 ms) in the WM group respectively. The ERP mean amplitude measures for P1 and N1 were submitted separately to three-way ANOVAs that examined group (WM, mere-repeat) × distractor item (match, mismatch) × hemisphere (ipsilateral, contralateral), and group was a between-subject factor.

#### Standardized low resolution electrical tomography analysis

As the ERP difference between match and mismatch condition was only found in the WM group, sLORETA analysis was only used in this group. On the basis of the ERP components’ scalp topographies, sLORETA (Pascual-Marqui, 2002) was used to localize the cortical generators of P1 and N1. sLORETA provides a unique standardized distributed linear solution to the inverse problem based on the neurophysiological assumption that the activities of neighboring cortical areas are coherent. Accordingly, it estimates multiple simultaneously active sources, thus avoiding the difficulties of estimating the number and position of the underlying dipoles. sLORETA uses a three-shell spherical head model co-registered to the MNI152 template, restricting solution space to the gray matter and hippocampus. The solution space is further partitioned to 6239 voxels at a 5-mm spatial resolution. With a transformation matrix, the standardized current density at each voxel is calculated, forming a voxel-based whole-brain (gray matter and hippocampus) sLORETA image. LORETA methods (including LORETA, sLORETA, eLORETA) have received considerable validation from studies combining them with other more precise localization methods such as functional Magnetic Resonance Imaging.

For the present study, the transformation matrix was calculated with a regularization parameter (smoothness) corresponding to a signal-to-noise ratio (SNR) of 10. Then the distributed neural activities for P1 (from 90 to 130 ms) and N1 (from 160 to 200 ms) were estimated individually for each condition. The resulting individual current density images for each condition were averaged across participants to obtain the final grand mean P1 and N1 sLORETA images. Voxels of the grand mean sLORETA images that showed maximal activities in each condition for P1 and N1 were located in anatomical regions and Brodmann areas (BAs).

#### Time–frequency analysis

EEG data were preprocessed using EEGLAB (Delorme and Makeig, 2004). In the WM group, all visual search trials, in which the responses were correct and the responses of the memory test task were correct, were selected for the following analysis. In the mere repetition group, the correct responses for visual search trials were selected for the further analysis. Continuous EEG data were band-pass filtered between 0.01 and 30 Hz. EEG epochs were segmented in 1400 ms time-windows (600 ms pre-stimulus and 800 ms post-stimulus) and baseline corrected using the pre-stimulus time interval. Trials contaminated with EOG artifacts (mean EOG voltages exceeding ±80 μV) or those with artifacts due to amplifier clipping, bursts of electromyographic (EMG) activity, or peak-to-peak deflections exceeding ±80 μV were excluded from analysis.

After all EEG data were reprocessed, an estimate of the oscillatory power as a function of time and frequency (time-frequency representation) was obtained from single-trial EEG epochs using the continuous Morlet wavelet transform (CWT) conducted by Letswave software^1^ (Mouraux and Iannetti, 2008). The parameters of central frequency (*ω*) and restriction (*σ*) in CWT were 5 and 0.15 respectively, and time-frequency representations were explored between 1 to 30 Hz in steps of 0.58 Hz. Single-trial time-frequency representations were then averaged to obtain the averaged time-frequency representations of every participant under each condition. The resulting averaged time-frequency representations were exported from Letswave and imported into MATLAB for further detailed analysis.

To analyze the power modulation of ongoing EEG rhythms after visual search stimuli onset, ERSP was calculated for every time-frequency pixel in the time-frequency representations. For each estimated frequency, ERSP was calculated as an increase or decrease of oscillatory power relative to the baseline interval (−500 ms to −100 ms) according to the formula: ER*_t, f_* % = [*A_t, f_* − *R_f_*]/*R_f_*, where *A_t, f_* was the signal power at a given time (*t*) and frequency (*f*), and *R_f_* was the averaged signal power of frequency f within the baseline interval (Pfurtscheller and Lopes da Silva, 1999). To avoid edge effects when performing CWT, the pre-stimulus time interval (−500 ms to −100 ms) was used as a baseline interval. After transforming the original power values to ERSP in the time-frequency representations, we performed an exploratory data-driven analysis routine to identify all the time-frequency regions of interest (TF-ROIs) which were most likely significantly modulated by the factors of distractor item and the corresponding spatial regions of interest (S-ROIs).

The exploratory data-driven analysis routine was performed as follows:

1. We first roughly identified several TF-ROIs with maximal modulations related to the match and mismatch condition. We achieved this by calculating the time-frequency difference maps corresponding to the match and mismatch condition across all the electrodes, and then the TF-ROIs, showing the largest modulation of each effect from the difference maps, were identified.
2. We calculated the mean of all the time-frequency pixels included in a specific TF-ROI for each electrode. For every TF-ROI, a paired-sample *t*-test (two-tailed) was performed for each electrode and the resulting *t*-value for the specific effect corresponding to this TF-ROI was extracted. Then all of the extracted *t*-values corresponding to the electrodes were plotted as a scalp map. Based on the scalp regions showing the most pronounced *t*-values, two S-ROIs which were related to corresponding effects were identified: the left occipito-parietal [(PO3 + PO7 + O1)/3] and right occipito-parietal [(PO4 + PO8 + O2)/3] regions. Based on the defined S-ROIs, we calculated the magnitude difference between match and mismatch conditions [expressed in ER% of (match-mismatch)] to evaluate the potential main effect of distractor item.
3. For each obtained time-frequency representation of the ERSP magnitude difference, we tested whether and when the resulting ERSP magnitudes in the post-stimulus interval were significantly different from the ERSP magnitudes in the pre-stimulus interval using a boot-strapping method (Delorme and Makeig, 2004; Durka et al., 2004). At each time-frequency point in the post-stimulus interval, investigated populations and reference populations were collected from 16 participants. The null hypothesis was that there was no mean difference between these two populations. The pseudo-*t*-statistic between the two populations was calculated, and we estimated the probability distribution of the pseudo-*t*-statistic by sampling with two replacement populations of the same size from the reference population. The permutation was executed 5000 times. The distributions of the pseudo-*t*-statistics from the reference population and the bootstrap *p*-value for the null hypothesis were generated.
4. This procedure yielded time–frequency distributions in which the brain responses within the post-stimulus interval were significantly different from the responses in the reference interval (Hu et al., 2012; Peng et al., 2012). To address the problem of multiple comparisons, the significance level (*p*-value) was corrected using a false discovery rate (FDR) procedure (Durka et al., 2004). In addition, to control for false-positive observations (Maris and Oostenveld, 2007), significant TF-ROIs were defined based on the following two criteria: (1) the time–frequency pixels were significantly different from the pre-stimulus interval at *p* < 0.05; (2) the time–frequency pixels had to include more than 125 consecutive significant time points (250 ms; Hu et al., 2013); and (3) Frequencies below 4 Hz (Delta-band) were not considered for oscillations as such an extremely low frequency band is often subject to artifacts due to sweating, movement and electrode drift (He and Raichle, 2009).

After TF-ROIs and S-ROIs were identified, we calculated the mean magnitude within the TF-ROIs at corresponding S-ROIs for each condition in WM group and mere repetition group, respectively. The resulting values were entered into a three-way repeated-measure ANOVA with the factors of group (WM, mere-repeat), distractor item (match, mismatch) and hemisphere (left, right).

## Results

### Behavioral measures

**Figure 2:**
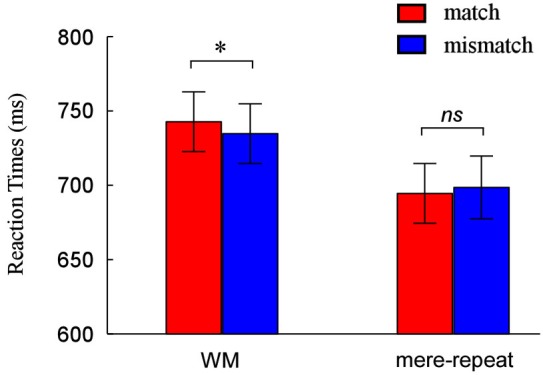
**Reaction times (RTs) and error rates as a function of match and mismatch condition when the cue was held in WM and when it was merely identified**. * *p* < 0.05, ** *p* < 0.01, *** *p* < 0.001.

Performance was accurate in both the search task (mean 97% correct for WM trials and mean 97% correct for priming trials) and memory task (96% correct, WM condition only). In the mere repetition group, responses on search trials were withheld as instructed (mean 97% correct). We analyzed median RTs of the correct responses in all tasks, using a 2(group: WM, mere-repeat) × 2(distractor item: match, mismatch) repeated-measures ANOVA. The interaction between group and distractor item was significant (*F*_(1,33)_ = 4.50, *p* < 0.05). There is a reliable difference between match and mismatch conditions in the WM task (*F*_(1,33)_ = 3.91, *p* < 0.05) and not in the priming task (*F*_(1,33)_ = 1.07, *p* = 0.31). Figure 2 depicts this pattern of performance.

### ERP measures

#### P1 component

**Figure 3:**
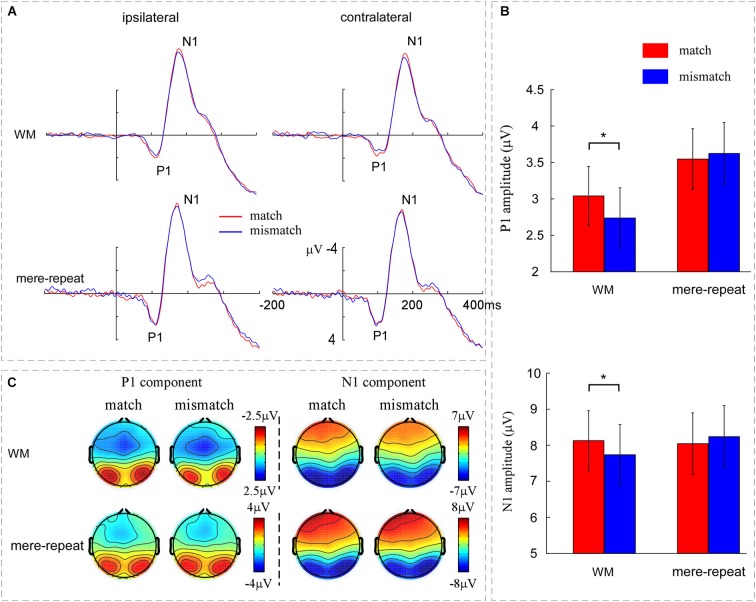
**(A)** The grand averaged ERPs across the WM group and the mere-repeat group for the match and mismatch conditions for distractor at contralateral vs. ipsilateral electrode sites. The left occipito-parietal electrodes include O1, PO3 and PO7 and the right occipito-parietal electrodes include O2, PO4 and PO8. **(B)** The mean amplitudes of P1 and N1 components across the WM group and the mere-repeat group for the match and mismatch conditions. The representative electrodes for P1 and N1 were O1, PO3, PO7, O2, PO4, and PO8. **(C)** The scalp voltage distribution maps of the P1 and N1 components across the WM group and the mere-repeat group for match and mismatch conditions. The time points of the P1 and N1 components were 90–130 ms and 160–200 ms in the WM group and 80–120 ms and 150–190 ms in the mere-repeat group, respectively. The color bars show the voltage value (in μV) of the components.

A three-way ANOVA with group (WM, mere-repeat), distractor item (match, mismatch) and hemisphere (ipsilateral, contralateral) was conducted on the P1 amplitude. There was a significant interaction effect between group and distractor item (*F*_(1,33)_ = 3.63, *p* < 0.05). In the WM group, the difference between match and mismatch condition was significant (*F*_(1,33)_ = 4.15, *p* < 0.05). P1 amplitude was larger in match condition (3.04 ± 0.40 μV) than in the mismatch condition (2.73 ± 0.41 μV). In the mere-repeat group, the difference between match and mismatch was not significant (*F*_(1,33)_ = 0.25, *p* = 0.62). No other main effects or interactions were significant (*p* > 0.10). For the P1 latency, the main effect of group was significant (*F*_(1,33)_ = 5.13, *p* < 0.05), indicating that the latency of WM group (109 ± 2 ms) was longer than mere-repeat group (102 ± 2 ms). The main effect of hemisphere was significant (*F*_(1,33)_ = 28.59, *p* < 0.01), indicating that the P1 latency was longer in ipsilateral (107 ± 1 ms) than in contralateral (103 ± 2 ms). No other significant main effects or interactions were observed. Figure 3 depicts this pattern of performance.

#### N1 component

Average peak amplitude measures from the electrodes of interest for N1 were submitted to an ANOVA with group (WM, mere-repeat), distractor item (match, mismatch) and hemisphere (ipsilateral, contralateral) as factors. The interaction effect between group and distractor item was significant (*F*_(1,33)_ = 4.50, *p* < 0.05. A further breakdown of the interaction showed a reliable difference between match and mismatch conditions in the WM group (*F*_(1,33)_ = 4.63, *p* < 0.05) and not in the mere-repeat group (*F*_(1,33)_ = 1.05, *p* = 0.31). In the WM group, the match between distractor and WM content elicited a larger (i.e., more negative) amplitude (−8.12 ± 0.82 μV) than the mismatch between them (−7.73 ± 0.83 μV). The main effect of hemisphere was significant (*F*_(1,33)_ = 24.40, *p* < 0.01), indicating that the N1 in the ipsilateral (−8.26 ± 0.60 μV) was more negative than contralateral (−7.80 ± 0.59 μV). No other main effects or interactions were significant (*p* > 0.10; see Figure 3). For the N1 latency, the main effect of group was significant (*F*_(1,33)_ = 27.36, *p*s < 0.01), indicating that the latency of WM group (178 ± 2 ms) was longer than mere-repeat group (165 ± 2 ms). The main effect of hemisphere was significant (*F*_(1,33)_ = 9.41, *p* < 0.01), indicating that the N1 latency was longer in ipsilateral (173 ± 1 ms) than in contralateral (170 ± 1 ms). No other main effects or interactions were significant (*p*s > 0.10). Figure 3 depicts this pattern of performance.

### Source localization

**Figure 4:**
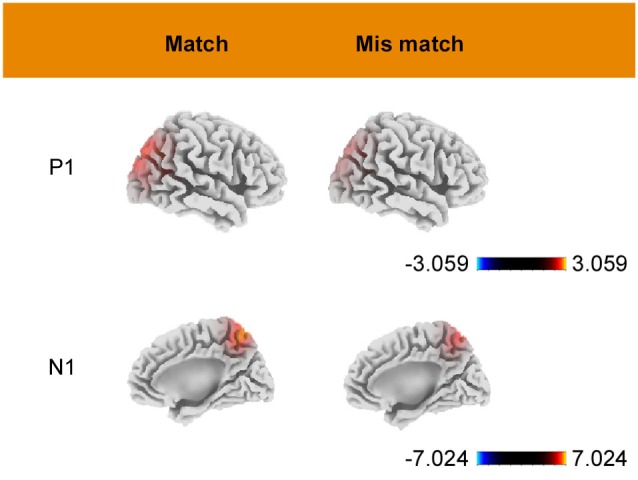
**Grand mean sLORETA images of P1 (90–130 ms) and N1 (160–200 ms) for match and mismatch conditions**. Color bars represent voxel current density values (A/m2).

Estimates of the underlying cortical generators obtained using sLORETA are displayed in Figure 4. As shown in the figure, P1-related activation in both conditions was mainly located at the superior occipital gyrus (BA 19, peak activation 35, −85, 30). N1-related activation, for the match condition, was located at a portion in the parietal lobe (BA7, peak activation −10, −60, 60). Under the mismatch condition, the maximum activition was located at the superior parietal lobule (BA7, peak activation −15, −65, 60). All of the above activations had a bilateral feature and only the coordinates of area with maximal activation are reported.

### Time-frequency results

**Figure 5:**
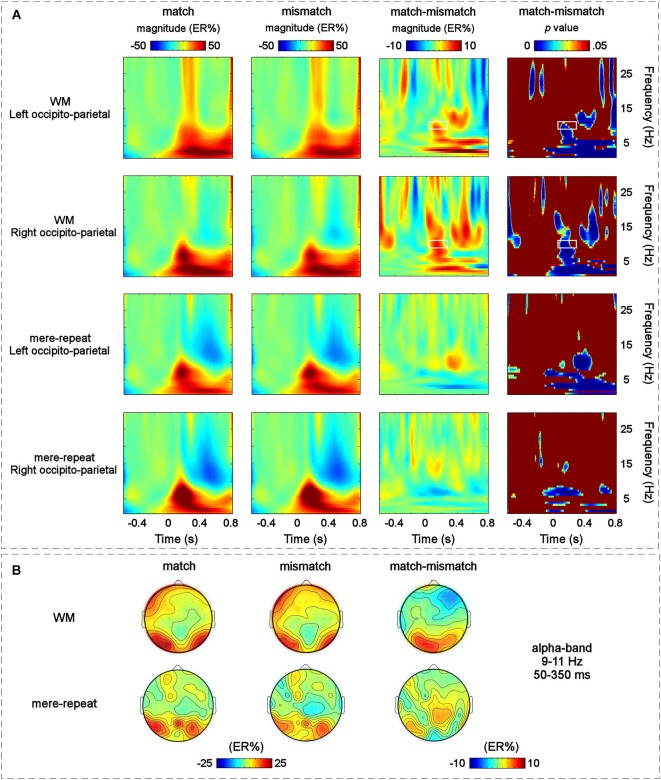
**(A)** The grand-average time-frequency representations for the match and mismatch conditions and the difference between them in the two S-ROIs across the WM group and the mere-repeat group: the left occipito-parietal [(PO3 + PO7 + O1)/3] and the right occipito-parietal [(PO4 + PO8 + O2)/3] regions. Each row corresponds to one S-ROI corresponding to the largest modulation of the specific effects. From left to right: the grand-averaged time-frequency representation for the match condition in two S-ROIs across the two group; the grand-averaged time-frequency representation for the mismatch condition in two S-ROIs across the two group; the grand-averaged time-frequency representation for the magnitude difference between the match and mismatch conditions in two S-ROIs across the WM group and the mere-repeat group and the results of corresponding bootstrapping statistical analyses at the significance level of *p* < 0.05 (FDR corrected). The time–frequency pixels displaying significant differences in ERSP magnitude (expressed as ER%) between the match and mismatch conditions are outlined by white rectangles (alpha-band, 9–11 Hz, 50–350 ms). Note that a pre-stimulus interval from −500 ms to −100 ms was used for the baseline. X-Axis, time (s); Y-axis, frequency (Hz). **(B)** The scalp topographies of the ERSP magnitudes (expressed as ER%) for the match and mismatch conditions and their differences within the defined TF-ROI across the two group.

The modulation of the main effect of the distractor item (match, mismatch) happened mainly in the left occipito-parietal and right occipito-parietal regions. The grand-average time-frequency representations of the two groups (WM group and mere repetition group) and the difference time-frequency representation between the WM group and mere repetition group (match-mismatch) in the left occipito-parietal and right occipito-parietal regions are illustrated in Figure 5A. A TF-ROI in the alpha-band (9–11 Hz, 50–350 ms) that showed the most pronounced WM guidance-related effect was defined (the area identified with a retangle in Figure 5A, *p* < 0.05, FDR corrected). The scalp topographies of ERSP magnitudes for the WM group and mere repetition group and the difference between the WM group and mere repetition group (match-mismatch) within the defined TF-ROI (9–11 Hz, 50–350 ms) are illustrated in Figure 5B. The mean ERSP magnitudes within the defined TF-ROI for the two conditions were submitted to a three-way within-subjuects repeated-measures ANOVA with group (WM, mere-repeat), distractor item (match, mismatch) and hemisphere (left, right) as factors. The results revealed a significant interaction effect between the group and the distractor item (*F*_(1,33)_ = 4.27, *p* < 0.05). Notably, the alpha-band ERS was significantly stronger for the match condition than for the mismatch condition only in the WM group (*F*_(1,33)_ = 3.56, *p* < 0.05) and not in the mere-repeat group (*F*_(1,33)_ = 1.10, *p* = 0.30). No other main effects or interactions were significant.

## Discussion

Using a modified delayed-to-match-sample task where subjects had to retain an item in visual memory, the present study tried to examine the temporal course of the WM guidance effect on attention. The RT measurements showed that the memory guidance (match minus mismatch) was significant under the normal experimental condition, namely that holding a representation in visual WM automatically biased the visual system to selectively process objects that matched that representation. Further, the electrophysioligical measurements indicated that the relation between the memory item and the search display also influenced the early compents of visual ERP, the P1 and N1 component. In contrast, neither behavioral effect nor electrophysioligical measurements effect of cue was found when the cue had to be identified but not held in memory. Therefore, only when the “template” held in visual WM was able to modulate the neural activity of the early visual cortex in a top-down manner, biasing attention to items that matched the contents of WM automatically.

The main result of the present study relates to the earliest WM guidance effect of cortical activity in the posterior P1 component (peak at approximately 110 ms after stimulus onset). The P1 amplitude was larger when the distractor stimulus matched the WM item, compared with when they did not match. It is well established that neural activity associated with WM guidance can be present early in the perceptual processing stage (Luck et al., 1993; Mangun et al., 1993; Wijers et al., 1997; Luck and Hillyard, 2000; Fu et al., 2005; Wang et al., 2012), as both P1 and N1 components are typically enlarged in amplitude when probes are presented to the memory-matched sapatial location. Jha (2002) also reported that such early attention modulation was supported by the occipito-temporal cortex. Although much of this data was generated from spatial WM studies, we provide further evidence that visual WM also results in modulation at early stages in visual processing.

Our P1-related sLORETA results suggested that visual WM guidance occurred in the occipital lobe at about 110 ms after stimulus onset, consistent with the source localization of P1 to the visual association cortex (Corbetta et al., 1990; Soto et al., 2005; Schoenfeld et al., 2007). Abundant research suggests that the feature WM-based network involves the fronto-temporal-occipital regions, and the reapprearance of a stimulus held in WM enhances activity in those areas (Soto et al., 2007, 2012b). After a visual object is stored in memory, neurons in the prefrontal cortex carry signals related to the WM stimulus and feed back to the visual cotex to enhance the activity of the neurons that code memory-relevant features and promote the selection of matching viusal items during later search (Olivers et al., 2011). In the present study, a match between a distractor and a WM representation was associated with increasead P1 amplitude in the occipital cortex. Thus, it is supposed that the P1 enhancement reflects the WM guidance effect, and this is also the first study to find such effect in the early visual cortex of human.

Interestingly, our evidence that automatic guidance of attention by the WM contents on initial perceptual representations seems to be in disagreement with some other studies in which WM effects were only found on the later N2pc and P3 components (Kumar et al., 2009; Telling et al., 2010). The critical factor here may be the experimental materials and task sets. As for the materials, the current task used pure visual figures as memory materials because they are difficult to verbalize. As 50 of them were generated, there was a low frequency of stimulus repetition and the shapes had a high level of inter-item similarity. Complex irregular pictures are more deeply processed in WM so as to elicit a strong guidance effect. In terms of task set, the major difference between the current task and above mentioned studies was that the distractor was peripheral, distant, and different from the target. Lavie (1995) indicated that the clear physical distinction between distractor and target is a key factor leading to early selection, so the WM content may affect the perceptual processing stage. Further, in previous studies, it should be noted that all distractors were presented together including memory-matching distractors, and they occupied the general area where the target could appear, capable of diluting distractor interference with each other. This dilution phenomenon, namely, the reduction of distractor interference with the addition of task-irrelevant distractor, is well documented in the literature (e.g., Brown et al., 1995; Roberts and Besner, 2005). That is, in the previous studies, the memory-match distractor was likely to have been diluted by other distractors because the representation of their features were highly activated in the process of searching for the target. Hence, the WM guidance has been confounded with the dilution of the memory-match distractor by other distractors. Therefore, the WM guidance effect was not always obvious. However, in the current experiment design, the distractor was the only stimulus appearing distant and different from the target, thereby exerting its maximal influence.

Moreover, it is noteworthy that the early WM guidance effect was not from the perceptual prime of WM content. In the present study, there was no evidence for bottom-up priming in P1 and N1 amplitude, suggesting that, the WM guidance effect from this source was weak. Similarly, Kumar et al. (2009) had observed the same result. This may reflect that perceptual prime is not enough to drive attention to a location where the item reappears.

In addition to the earliest, WM-driven P1 effects, there is also evidence that the reappearance of a WM item influenced the later N1 stage. Similar to the effect of the P1 component, the N1 amplitude was greater in the match trials relative to when the WM stimulus and the distractor were mismatched. N1 evidently indexes distinct attentional operations to P1 (e.g., Hillyard et al., 1998; Natale et al., 2006; Klimesch et al., 2007); for example, N1 has been attributed to index the engagement or orienting of attention to a task-relevant location (Luck et al., 1990; Natale et al., 2006). Luck et al. (1990) asked subjects to attend to the left or right hemifield of a visual display while fixating on a central point. Stimuli were presented to the left or right visual fields on separate trials (unilateral stimuli) or both fields simutaneously (bilateral stimuli). They proposed that the presentation of a unilateral stimulus on the unattended side may automatically attract attention away from the attended location briefly such that a subsequnent attended unilateral stimulus requires a re-orientation of attention back to the task relevant location, which results in an N1 attention effect. In the present study, when participants searched for a target letter, the memory-matching distractor may have immediately received attention priority and guided attention to the specific location. This is referred to as attentional capture, and is a well documented P1 effect. After that, through top-down volitional feedback signals that depend on the observation goal, participants realized that the attended item was a distractor rather than the target and then attention was re-oriented to the task-relevant location.

Previous studies have demonstrated that visual N1 amplitude is larger for attented-location stimuli than for unattended-location stimuli and the N1 wave reflects a discrimination process that is applied to the attended location (Vogel and Luck, 2000). Current source analyses provide further insights. N1-related sLORETA results suggested that N1 originates in the parietal lobe at about 180 ms after stimulus onset, a finding consistent with previous N1 source data (Di Russo et al., 2002, 2003). The parietal area is generally considered to belong to the top-down control network for spatial attention (Nobre et al., 1997; Corbetta, 1998). Recently Soto et al. (2012a) indicated that PPC and middle temporal (hippocampal) cortices may be involved in the strategic modulation of WM biases through expectations/foreknowledge about the incoming validity of WM items for visual selection goals, namely, boosting or suppressing WM biases when WM contents predict a target or a distractor (Soto et al., 2012a). Some other studies futher illustrated the role of the left parietal cortex in suppressing capture by salient items (Mevorach et al., 2010) or WM content (Soto et al., 2011, 2014). Our data are consistent with these suggestions and futher suggest that the superior parietal lobule may be involved in suppressing neural processing stemming from attention grabbing items that match the WM content. Though participants have known that the memory picture would never be the target, their attention is still driven to the memory-matching distractor. However, through suppressing the interference from task-irrelevent stimuli in the periphery, attention was re-oriented to the task-relevant target search and larger N1 amplitude corresponds to stronger suppression processing. This process relfects the cognitive control function of the parietal lobule. This proposal is further supported by evidence from time-frequency data.

In relation to frequency domain results concerning the visual search task, alpha-band ERSP increased significantly in the match condition compared with the mismatch condition. Stronger alpha-band ERSP magnitudes emerged in a relatively early period, extending from 50 to 350 ms, implying their potential involvement in an early perceptual processing period. In accordance with this, alpha power might reflect the inhibition of sensory (bottom up) processing areas via attentional (top-down) control mechanisms (Klimesch et al., 2007; Palva and Palva, 2007; Khader et al., 2010; Foxe and Snyder, 2011). A similar explanation has been provided by Jensen et al. (2002) and Jokisch and Jensen (2007), who suggested that “the inhibition or disengagement of occipital–parietal areas could serve to suppress input from the visual stream”. This alpha rhythm inhibition hypothesis is consistent with our results which showed that the occipito-parietal alpha-band oscillations increased in the match condition compared to mismatch condition. Following Jensen et al.’ line of reasoning, the memory-matching distractor might interfere with task-relevent processing. Therefore, the inhibition of visual areas might be a goal-driven control mechanism for shielding target-relevent information from distracting visual input, leading to an increase of alpha synchronization over the parietal–occipital cortex. Moreover, it is worth noting that the time-frequency pixels had to include more than 125 consecutive significant time points (0.25 s) (Hu et al., 2013). So the time-frequency presentation only reflects the inhibition effect for memory-matching distractor by reason of the time limitation.

To conclude, the current findings support the biased competition model of visual selection (Desimone and Duncan, 1995; Desimone, 1998). According to this framework, once an object is held in WM, it can automatically bias attention to the item whose features are preactivated in WM, even though the item is disruptive to the target search. The reappearance of a stimulus held in WM enhanced activity in the occipital areas, consistent with Soto et al. (2007), reflected by the P1 component. Combined with time-frequency data and the function of the parietal lobe (Soto et al., 2011), we also observed that this initial capture of attention by WM could be inhibited by competing visual inputs by attention re-orientation in the N1 stage, as reflected by the alpha-band ERSP. More empirical research is necessary to explore how cognitive control influences WM information at later response output stages.

## Conflict of interest statement

The authors declare that the research was conducted in the absence of any commercial or financial relationships that could be construed as a potential conflict of interest.

## Footnotes

^1^http://nocions.webnode.com/letswave/
